# RNA cytidine acetyltransferase NAT10 maintains T cell pathogenicity in inflammatory bowel disease

**DOI:** 10.1038/s41421-025-00781-5

**Published:** 2025-03-04

**Authors:** Haixin Li, Xuemin Cai, Changfen Xu, Xinhui Yang, Xiaohan Song, Yuxin Kong, Mei Yang, Qielan Wu, Song Guo Zheng, Yiming Shao, Ping Wang, Jing Zhou, Hua-Bing Li

**Affiliations:** 1 Tongji University Cancer Center, Shanghai Tenth People’s Hospital, School of Medicine, Tongji University, Shanghai, China; 2 Center for Immune-Related Diseases at Shanghai Institute of Immunology, Ruijin Hospital, Shanghai Jiao Tong University School of Medicine, Shanghai, China; 3 Shanghai Jiao Tong University School of Medicine-Yale Institute for Immune Metabolism, Shanghai Jiao Tong University School of Medicine, Shanghai, China; 4 Institute of Immunological Innovation and Translation, Chongqing Medical University, Chongqing, China; 5 Department of Gastroenterology, Ruijin Hospital, Shanghai Jiaotong University School of Medicine, Shanghai, China; 6 Department of Ophthalmology, Shanghai Key Laboratory of Orbital Diseases and Ocular Oncology, Ninth People’s Hospital, Shanghai JiaoTong University School of Medicine, Shanghai, China; 7 Department of Rheumatology & Immunology, School of Cell and Gene Therapy, Songjiang Research Institute, Songjiang Hospital Affiliated to Shanghai Jiao Tong University School of Medicine, Shanghai, China; 8 The Key Laboratory of Sepsis Translational Medicine, Guangdong Medical University; Dongguan Key Laboratory of Sepsis Translational Medicine, The First Dongguan Affiliated Hospital, Guangdong Medical University, Dongguan, Guangdong China; 9 Department of Geriatrics, Medical Center on Aging of Ruijin Hospital, Shanghai Jiao Tong University School of Medicine, Shanghai, China; 10 Chongqing International Institute for Immunology, Chongqing, China

**Keywords:** Immunology, Cell death, RNA modification

## Abstract

The emerging field of epitranscriptomics is reshaping our understanding of post-transcriptional gene regulation in inflammatory diseases. *N*^4^-acetylcytidine (ac^4^C), the only known acetylation modification in RNA catalyzed by *N*-acetyltransferase 10 (NAT10), is known to enhance mRNA stability and translation, yet its role in inflammatory bowel disease (IBD) remains unclear. In this study, we discovered that *Nat10* expression correlates with inflammatory and apoptotic pathways in human ulcerative colitis CD4^+^ T cells. Our further analysis revealed that the deficiency of NAT10 led to a disruption of T cell development at steady state, and identified a pivotal role for NAT10 in preserving the pathogenicity of naïve CD4^+^ T cells to induce adoptive transfer colitis. Mechanistically, the lack of NAT10 triggers the diminished stability of the anti-apoptotic gene BCL2-associated athanogene 3 (*Bag3*), initiating a cascade of events that includes the upregulation of apoptosis-related genes and an accelerated rate of apoptosis in T cells. Our findings reveal a previously unrecognized role of the NAT10-ac^4^C-*Bag3* axis in preserving T cell balance and suggests that targeting RNA ac^4^C modification could be a promising therapeutic approach for IBD.

## Introducion

Inflammatory bowel disease (IBD) arises from a sophisticated interaction of genetic, environmental, and immunological elements, impacting an approximated 7 million patients worldwide^1^. The imbalance in T cell immune responses significantly promotes the progression of IBD, underscoring the need for a more profound investigation into the molecular mechanisms that regulate T cell behavior as the disease evolves. In recent years, the role of RNA modifications in epigenetic regulation has garnered significant attention. There are over 170 types of RNA modifications constituting the epitranscriptome^2^. Research has found that the epitranscriptome expands the regulatory functions of RNA by affecting various aspects of RNA metabolism, thereby opening up new avenues for gene regulation^2–4^. Previous investigations have underscored the importance of RNA modifications in regulating T cell immunity^5–11^. Nonetheless, the majority of these studies have concentrated on the realm of RNA methylation, leaving a void in our knowledge regarding other forms of RNA modification. Among these, *N*^4^-Acetylcytidine (ac^4^C), unique as the only known acetylation process in RNA, has emerged as a highly conserved modification in eukaryotic RNA2,^2,12,13^, playing a pivotal role in diverse cellular processes. However, the involvement of ac^4^C in the development and function of immune cells is still unclear.

*N*-acetyltransferase 10 (*Nat10*), originally identified as a lysine acetyltransferase (KAT), is now universally recognized as the exclusive enzyme responsible for the acetylation of ac^4^C on mRNA^12,14,15^. NAT10-mediated ac^4^C acetylation has been observed to regulate various post-transcriptional processes, including enhancing mRNA stability and translation^12^, maintaining tRNA conformation^15,16^, and aiding in ribosome maturation^14,17^, thus regulating human embryonic stem cell (hESC) fate transitions^18^, tumorigenesis^19,20^, and human accelerated aging syndrome^21,22^. Nevertheless, the role of ac^4^C in modulating T cell homeostasis and its pathogenic effects in the context of IBD is yet to be identified.

Here, we found that *Nat10* levels are associated with the activation of inflammatory and apoptotic pathways within CD4^+^ T cells in patients with IBD. To further investigate its role, we generated mice with lineage-specific deletion of NAT10 in T cells and found that *Nat10*-deficient mice exhibited a pronounced decline in both proportion and number of CD4^+^ and CD8^+^ T cells under steady state in peripheral immune organs compared to wild-type (WT) mice. Besides, *Nat10*-deficient naïve CD4^+^ T cells failed to induce adoptive transfer colitis. After analyzing the RNA-seq data, we found a significant enrichment of genes related to the apoptotic pathway in *Nat10*-deficient CD4^+^ T cells. Subsequent experiments demonstrated that *Nat10* could directly bind to the mRNA encoding the anti-apoptotic factor BCL2-associated athanogene 3 (*Bag3*), thereby maintaining its stability. The absence of NAT10 elicits the downregulation of the *Bag3* gene, setting off a series of downstream events that include the upregulation of genes associated with apoptosis and an escalated apoptotic rate of T cells. Our research unveils a previously unknown function of the NAT10-ac^4^C-*Bag3* axis in preserving the homeostatic balance of T cells.

## Results

### *Nat10* expression correlates with inflammatory and apoptotic pathways in human ulcerative colitis CD4^+^ T cells

**Fig. 1:**
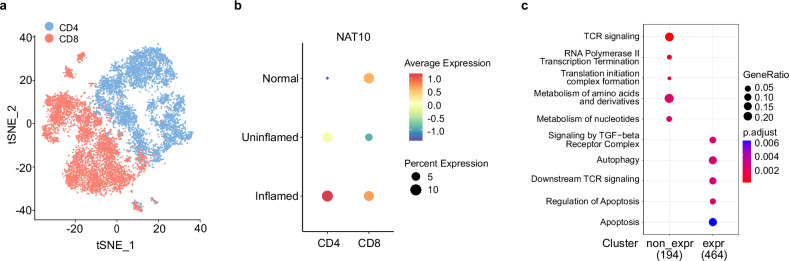
*Nat10* expression in CD4^+^ T cells links to inflammation and apoptosis signaling in human ulcerative colitis. **a** The *t*-distributed Stochastic Neighbor Embedding (*t*-SNE) visualization of previously published scRNA-seq data shows distinct clustering of colonic T cells in patients with ulcerative colitis (GEO accession: GSE235665)^23^. **b** The dot plot displays the expression of *Nat10* in CD4^+^ and CD8^+^ T cells across endoscopically inflamed, uninflamed, and normal colonic tissue samples (GEO accession: GSE235665)^23^. The color scale represents the average expression levels, with warmer colors indicating higher expression. The size of the dots corresponds to the percentage of cells expressing *Nat10*, with larger dots indicating a higher percentage. **c** The REACTOME enrichment analysis compares genes associated with *Nat10*-expressing and non-expressing CD4^+^ T cells (GEO accession: GSE235665)^23^. The *x*-axis indicates the presence (expr group) or absence (non_expr group) of *Nat10* expression, while the *y*-axis lists various REACTOME pathways. The dot color reflects the enrichment significance, and the dot size represents the gene ratio, showing the degree of gene enrichment within the pathways.

IBD, encompassing Crohn’s disease and ulcerative colitis (UC), is characterized by chronic inflammation of the gastrointestinal tract, with T cells playing a pivotal role in the pathogenesis. To explore the role of NAT10-mediated ac^4^C acetylation in regulating T cell activity during IBD, we analyzed previously published single-cell transcriptomes of sorted T cells isolated from inflamed and non-inflamed colonic tissues of patients with UC, and colonic T cells from healthy subjects and patients with UC in remission as control groups^23^. The T cells were effectively clustered into CD4^+^ and CD8^+^ populations (Fig. 1a). Our analysis revealed that, in comparison with samples from healthy tissues, *Nat10* expression was diminished in CD8^+^ T cells from non-inflamed colonic tissue and remained largely unchanged in CD8^+^ T cells from inflamed tissues (Fig. 1b). However, in CD4^+^ T cells, *Nat10* expression was notably elevated in non-inflamed colonic tissues and exhibited a substantial increase in inflamed tissues (Fig. 1b). These results suggest that NAT10-mediated ac^4^C modification in CD4^+^ T cells may play a role in the progression of UC. We further explored the single-cell sequencing (scRNA-seq) data and found that in CD4^+^ T cells lacking *Nat10* expression, there was a significant enrichment of highly expressed genes associated with apoptosis-related signaling pathways (Fig. 1c), implying that *Nat10* expression in CD4^+^ T cells links to the regulation of apoptosis in human UC.

### *Nat10* expression is selectively enhanced in response to T cell activation

**Fig. 2:**
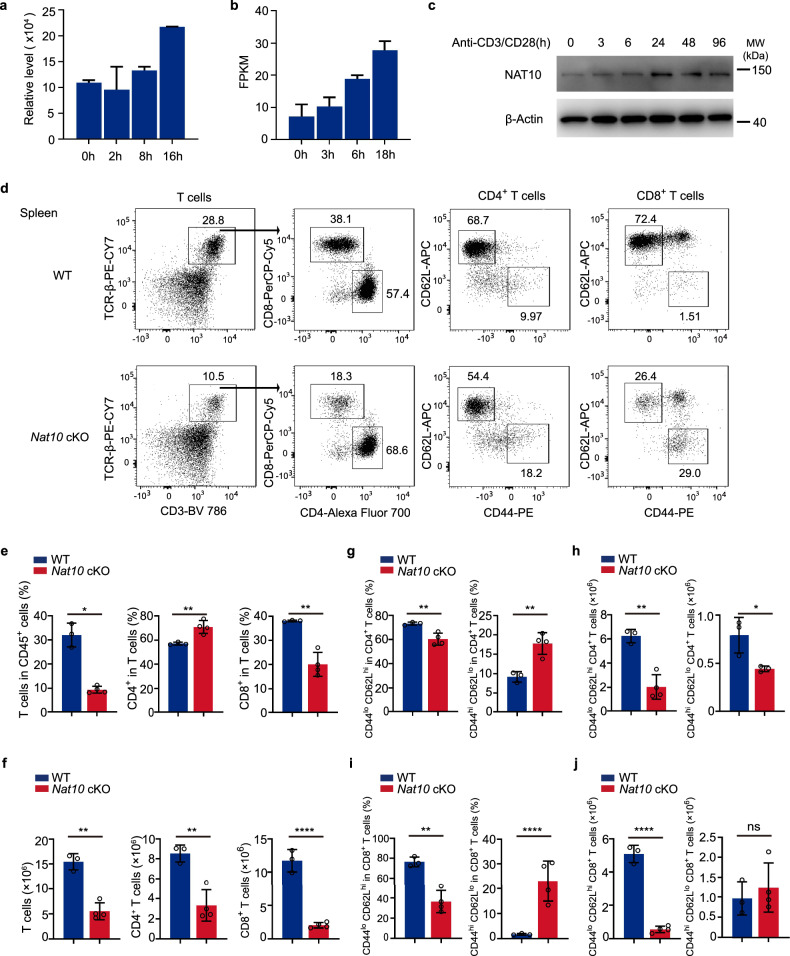
T cell-specific deletion of *Nat10* leads to disrupted splenic T cell homeostasis. **a** NAT10 protein expression was analyzed by a previously published proteomics dataset (PRIDE accession: PXD004367 and PXD005492)^24^. **b** *Nat10* mRNA expression was analyzed by our previously published RNA-seq dataset (GEO accession: GSE184909) ^7^. **c** Naïve CD4^+^ T cells were stimulated with anti-CD3/CD28 antibodies for 0, 3, 6, 24, 48, and 96 h. NAT10 protein expression was assessed by western blot. Data represent one of three independent experiments. **d** Representative dot plots illustrate the percentages of CD4^+^ and CD8^+^ T cells in the spleens of WT and *Nat10* conditional knockout (cKO) mice. Expression of CD62L and CD44 on splenic T cells from WT and *Nat10* cKO mice is also shown. Numbers in the quadrants indicate the percentage of cells in each subset. **e**, **f** The ratios (**e**) and absolute numbers (**f**) of various T cell subsets in the spleens of WT and *Nat10* cKO mice are displayed (*n* = 3–4). **g**, **h** The ratios (**g**) and absolute numbers (**h**) of CD44^lo^CD62L^hi^ and CD44^hi^CD62L^lo^CD4^+^ T cells in the spleen are shown (*n* = 3–4). **i**, **j** The ratios (**i**) and absolute numbers (**j**) of CD44^lo^CD62L^hi^, and CD44^hi^CD62L^lo^CD8^+^ T cells in the spleen are depicted (*n* = 3–4). Data in **d**–**j** represent one of three independent experiments and are shown as mean ± SEM. Statistical significance was determined using unpaired Student’s *t*-test: *p < 0.05, **p < 0.01, ****p < 0.0001. NS, not significant.

Previous studies have highlighted the dynamic nature of RNA modification-related genes during T cell activation^5–7^. However, these studies mainly focus on RNA methylation and largely remain unknown about other RNA modifications, especially the only RNA acetylation-ac^4^C that is catalyzed by *Nat10*. This has prompted us to delve deeper into the significance of *Nat10*’s role throughout this process. Therefore, we performed a systematic quantification of *Nat10* expression during T cell activation^7,24^, and observed a gradual increase in both protein and RNA expression at various time points post-activation (Fig. 2a, b). This initial observation was intriguing and suggests a potential correlation between *Nat10* expression and the progression of T cell activation.

To validate these findings and further explore the temporal dynamics of *Nat10* expression, we conducted an in vitro experiment. We isolated naïve CD4^+^ T cells from peripheral lymphoid organs of WT mice. These cells, representing the initial, unactivated state of T cells, were then stimulated with anti-CD3/CD28 antibodies, which are known to mimic the natural activation signals that T cells receive during an immune response. The activation was carried out at several time points: 0, 3, 6, 24, 48, and 96 h, allowing us to capture the early and late stages of T cell activation. Following the activation, we purified the proteins from these time-point samples and performed western blot analysis to assess the protein expression levels of NAT10 (Fig. 2c). The results were consistent with our initial findings, showing that NAT10 expression starts from a basal level in the naïve state and then increases progressively over time. This expression pattern implies that *Nat10* might play a role in modulating T cell function.

### *Nat10* is not essential for thymic T cell development

To further elucidate the physiological role of *Nat10* in T cells, we conducted a detailed investigation by employing a conditional gene knockout strategy. This strategy was designed to selectively remove *Nat10* in T cells, utilizing transgenic Cre recombinase that is driven by the regulatory regions of the mouse *Cd4* gene. Upon successful generation of the *Nat10*^flox/flox^ *Cd4*^Cre^ (cKO) mice and WT littermates (Supplementary Fig. S1a), we performed quantitative reverse transcription PCR (RT-qPCR) and western blot analysis to assess the levels of *Nat10* mRNA and protein respectively in CD4^+^ T cells, and found the expression of *Nat10* was effectively ablated in CD4^+^ T cells isolated from cKO mice, indicating that our conditional knockout approach was successful (Supplementary Fig. S1b, c).

To explore the consequences of *Nat10* deficiency on T cell development, we conducted a comparative analysis of T cell subsets in the thymus of WT and cKO mice. The thymus is the primary site where T cells mature and differentiate into various functional subsets. We examined the distribution and frequency of different T cell populations, including double-positive (DP) CD4^+^CD8^+^ and single-positive (SP) CD4^+^ or CD8^+^ T cells, as well as double-negative (DN) subsets, and observed that the absence of *Nat10* did not lead to any significant disruption in the developmental stages of T cells within the thymus (Supplementary Fig. S1d–f). These observations suggest that the lack of *Nat10* does not impede the latter stages of T cell maturation within the thymus.

### *Nat10* is crucial for preserving T cell homeostasis

Having established that *Nat10* does not affect thymic T cell development, we shifted our focus to examine the role of *Nat10* in the peripheral lymphatic system, which is crucial for T cell function and survival outside the thymic environment. We found that the proportion and number of total T cells were considerably decreased in the spleen and peripheral lymph nodes (mesenteric and inguinal lymph nodes) of cKO mice, compared to their WT counterpart (Fig. 2d–f and Supplementary Fig. S2). Further analysis revealed that the numbers of both CD4^+^ and CD8^+^ T cells were simultaneously reduced (Fig. 2d–f and Supplementary Fig. S2). We then assessed the activation status of T cells and found that both CD4^+^ and CD8^+^ T cells in the cKO mice were in a more activated state (Fig. 2d, g, i and Supplementary Fig. S2). However, due to the significant reduction in the absolute numbers of CD4^+^ and CD8^+^ T cells, the total number of cells at effector stages was decreased or unchanged (Fig. 2d–j). These findings indicate that the absence of *Nat10* has a detrimental effect on the maintenance of T cell populations in the periphery.

**Fig. 3:**
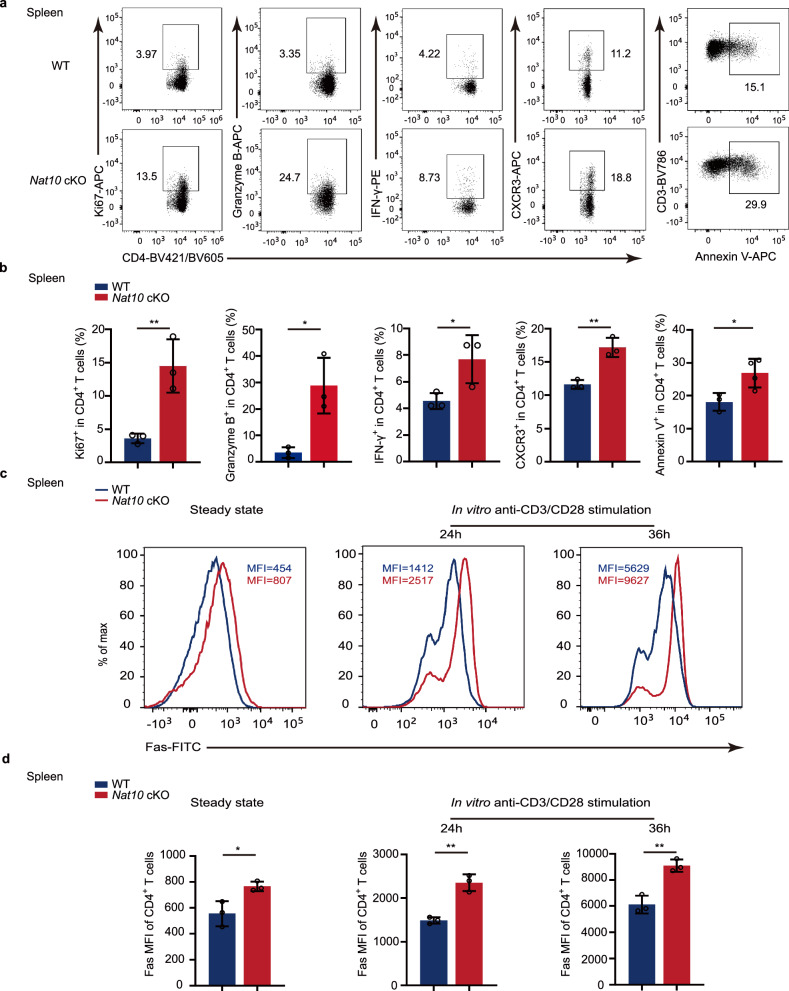
*Nat10*-deficient naïve CD4^+^ T cells show hyperactivation and increased apoptosis. **a** Characteristic dot plots illustrate the distribution of Ki67, Granzyme B, IFN-γ, CXCR3, and Annexin V-positive CD4^+^ T cells in the spleens of WT and *Nat10* cKO mice at a steady state. **b** Percentages of Ki67, Granzyme B, IFN-γ, CXCR3, and Annexin V-positive CD4^+^ T cells in **a** are shown (*n* = 3). **c** Characteristic flow cytometry histograms illustrate the distribution of Fas-positive CD4^+^ T cells from WT and *Nat10* cKO mice at a steady state or purified splenic naïve CD4^+^ T cells activated with anti-CD3/CD28 antibodies for 24 and 36 h. **d** Percentages of Fas mean fluorescence intensity (MFI) of CD4^+^ T cells in **c** are shown (*n* = 3). Data represent one of three independent experiments and are shown as mean ± SEM. Statistical significance was determined using unpaired Student’s *t*-test: *p < 0.05, **p < 0.01. NS, not significant.

In order to explore the cause of the reduced T cell number resulting from NAT10 deficiency, we examined the proliferation, apoptosis and migratory marker expression of T cells. Our findings indicated an increase in the proliferation (Ki67 expression) and migratory ability (CXCR3 expression) of CD4^+^ T cells when *Nat10* was absent (Fig. 3a, b and Supplementary Fig. S3a–d). However, we also observed a significant increase in the apoptosis (Annexin V expression) of CD4^+^ cells following the knockout of *Nat10* at steady state (Fig. 3a, b and Supplementary Fig. S3a–d). This enhanced apoptosis may be the principal reason for the decreased number of T cells in the absence of *Nat10*. Further analysis revealed that the absence of *Nat10* led to an increased expression of cytokines (granzyme B and IFN-γ) (Fig. 3a, b and Supplementary Fig. S3a–d). In addition, we also detected the levels of Fas expression, a marker of activation-induced cell death, in CD4^+^ T cells under steady state and after anti-CD3/CD28 antibodies stimulation. We found that, regardless of the state, *Nat10*-deficient CD4^+^ T cells expressed higher levels of Fas compared to WT cells (Fig. 3c, d and Supplementary Fig. S3e, f). These results collectively indicates that the absence of *Nat10* leads to a hyperactivated state of T cells.

### *Nat10* regulates the ability of CD4^+^ T cells to initiate spontaneous colitis

**Fig. 4:**
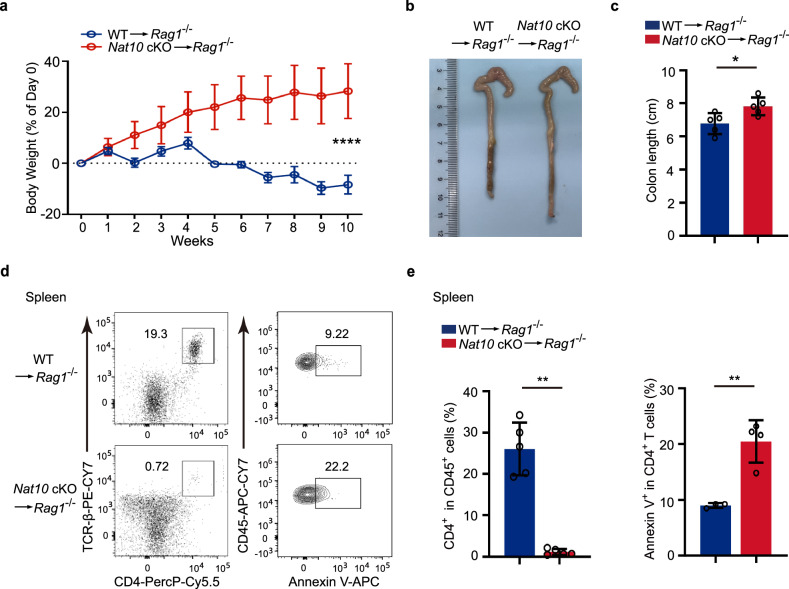
The absence of *Nat10* leads to an impaired ability of CD4^+^ T cells to induce adoptive transfer colitis. **a** Naïve CD4^+^ T cells (5 × 10^5^) were purified from either *Nat10* cKO mice or their WT counterparts and then transferred into *Rag1*^−/−^ mice. The body weight of the recipient mice was monitored weekly (*n* = 5) and statistically assessed using a two-way analysis of variance (ANOVA). **b** Colonic tissue was harvested from the mice described in **a** at 10 weeks post-adoptive transfer colitis induction, with representative images provided. **c** The colon length of the recipient mice was measured from the caecum to the proximal rectum in each group (*n* = 5) and analyzed using unpaired Student’s *t*-test. **d** Characteristic dot plots illustrate the composition and Annexin V expression of transferred CD4^+^ T cells in the spleen of recipient mice 10 weeks post-transfer. **e** The proportion of CD4^+^ T cells and percentage of Annexin V-positive CD4^+^ T cells as depicted in **d** (*n* = 3–5) were analyzed using unpaired Student’s *t*-test. Data represent one of three independent experiments and are shown as mean ± SEM. *p < 0.05, **p < 0.01. NS, not significant.

Adoptive transfer colitis serves as a well-established T cell-based model of inflammatory bowel disease^25,26^. We commenced by isolating naïve CD4^+^ T cells from both WT and *Nat10* cKO mice, and then transferred them separately into *Rag1*^−/−^ recipient mice, thereby inducing an adoptive transfer colitis model. Throughout the course of the experiment, we monitored the body weight of the recipient mice every week as a measure of their health and response to the transferred T cells. We found that the mice that received the WT naïve CD4^+^ T cells exhibited a noticeable weight loss during the process (Fig. 4a). This weight loss is indicative of the onset of colitis, a common manifestation in this model. In contrast, the mice that received the *Nat10*-deficient naïve CD4^+^ T cells not only maintained their weight but also exhibited a continued weight gain (Fig. 4a). Furthermore, these mice displayed a longer colon length compared to those injected with WT naïve CD4^+^ T cells (Fig. 4b, c), suggesting a potentially altered disease progression in the absence of *Nat10*.

To gain deeper insights into the long-term effects of *Nat10* deficiency on the transferred T cells, we analyzed the composition of donor CD4^+^ T cells 10 weeks post the induction of adoptive transfer colitis, and observed a significant difference in the persistence of these cells within the recipient mice. Specifically, the *Nat10* KO CD4^+^ T cells were largely depleted from the spleen and peripheral lymph nodes of the recipients compared with WT cells (Fig. 4d, e and Supplementary Fig. S4), indicating a possible role of *Nat10* in the survival and maintenance of naïve CD4^+^ T cells in vivo.

Further investigation into the underlying cause of this depletion revealed a pronounced increase in the apoptosis (Annexin V expression) of CD4^+^ T cells derived from *Nat10* cKO mice when compared to those from WT mice (Fig. 4d, e). This is also consistent with the increased apoptosis of *Nat10* KO cells under homeostasis (Fig. 3a, b). The heightened apoptosis in the absence of *Nat10* could be a critical factor contributing to the diminished presence of these cells in the lymphatic organs, thus reducing their capacity to induce adoptive transfer colitis. These findings imply the importance of *Nat10* in regulating T cell homeostasis and suggest its potential as a target for therapeutic intervention in immune-related disorders.

### *Nat10* deficiency caused the enrichment of genes in apoptosis-related pathways

**Fig. 5:**
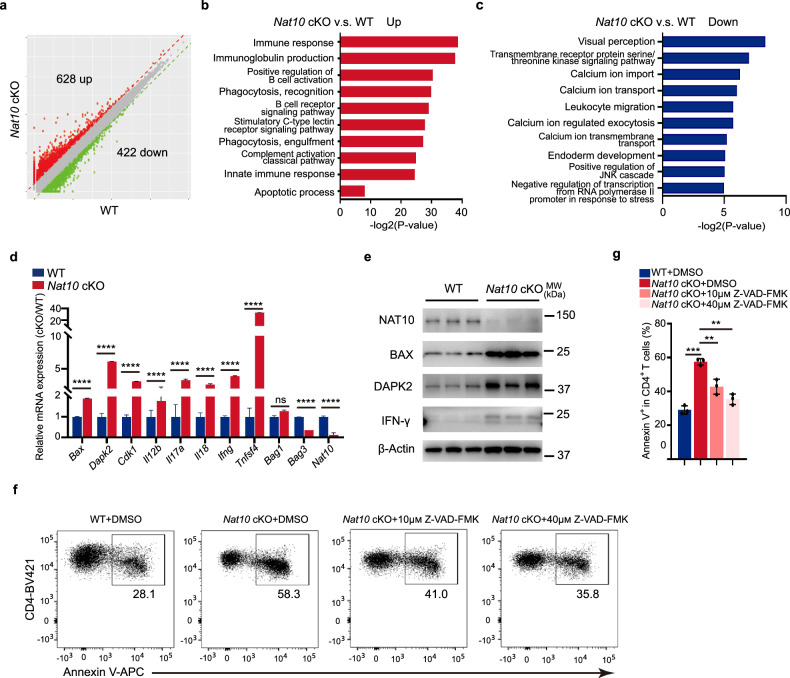
*Nat10* deficiency results in an upregulation of apoptosis-related genes in naïve CD4^+^ T cells. **a** The scatter diagram exhibits the differentially expressed genes (|fold change| > 2; *p* < 0.05; upregulated genes, red; downregulated genes, green) in *Nat10* cKO-derived naïve CD4^+^ T cells compared to WT-derived naïve CD4^+^ T cells. **b** Gene Ontology (GO) analysis of the upregulated genes according to the *p*-value derived from the RNA-seq data shown in **a**. **c** GO analysis of the downregulated genes according to the *p*-value from the RNA-seq data in (**a**). **d** Validation of gene expression of *Bax*, *Dapk2*, *Cdk1*, *Il12b*, *Il17a*, *Il18*, *Ifng*, *Tnfsf4*, *Bag1,* and *Bag3* via quantitative reverse transcription PCR (RT-qPCR) between WT and *Nat10* cKO naïve CD4^+^ T cells (*n* = 3). **e** Validation of protein expression of BAX, DAPK2 and IFN-γ via western blot between WT and *Nat10* cKO naïve CD4^+^ T cells. **f**, **g** The apoptotic rates of both WT and *Nat10* cKO naïve CD4^+^ T cells administrated with 10 and 40 μM Z-VAD-FMK, following 24-h stimulation with anti-CD3/CD28 antibodies, are illustrated in representative dot plots (**f**) and summarized in a graph (**g**) (*n* = 3). Data represent one of three independent experiments and are shown as mean ± SEM. Statistical significance was determined using unpaired Student’s *t*-test: **p < 0.01, ***p < 0.001, ****p < 0.0001. NS, not significant.

Given the phenotype observed in *Nat10* cKO mice, which includes disrupted T cell homeostasis and heightened apoptosis, we proceeded to explore the molecular underpinnings of these effects. We isolated naïve CD4^+^ T cells from WT and *Nat10* cKO mice for RNA-seq, and then established a fold-change threshold of greater than 2 and a *p* < 0.05 to identify genes that were differentially expressed. Our analysis unveiled a substantial shift in gene expression, with hundreds of genes exhibiting either increased or decreased expression levels in the *Nat10*-deficient naïve CD4^+^ T cells (Fig. 5a). Intriguingly, the predominantly upregulated genes were found to be linked to immune response mechanisms and the process of apoptosis (Fig. 5b), a pattern that aligns with the observations from human IBD samples (Fig. 1c). Conversely, the genes that were downregulated were notably enriched in biological pathways related to visual perception (Fig. 5c), suggesting a broader impact of *Nat10* deficiency on cellular functions beyond the immune system. To validate the RNA-seq findings, we conducted qPCR and western blot experiments and confirmed that the expression levels of genes known to promote apoptosis, such as *Bax* and *Dapk2*, were significantly elevated in *Nat10* KO naïve CD4^+^ T cells (Fig. 5d, e).

Considering the established role of *Nat10* in histone acetylation^18^, which is a key epigenetic modification influencing gene expression, we hypothesized that *Nat10* might also modulate chromatin accessibility. To test this, we performed assays for transposase-accessible chromatin using sequencing (ATAC-seq). However, we found that the genes proximal to regions with altered chromatin accessibility post-*Nat10* KO did not significantly overlap with the differentially expressed genes identified by RNA-seq. This observation suggests that *Nat10*’s influence on gene expression may not be mediated through changes in chromatin accessibility (Supplementary Fig. S5).

To ascertain whether the observed reduction in the number of *Nat10*-deficient naïve CD4^+^ T cells was indeed a consequence of increased apoptosis, we employed an in vitro treatment with Z-VAD-FMK, a potent and pan-caspase inhibitor^27^. Our results demonstrated that the heightened apoptosis observed in *Nat10*-deficient naïve CD4^+^ T cells could be effectively mitigated by this treatment (Fig. 5f, g). Thus, the deletion of *Nat10* leads to the upregulation of apoptosis-related genes of naïve CD4^+^ T cells without alterations in chromatin accessibility, indicating that *Nat10*’s role in regulating apoptosis may be independent of its effects on the epigenome.

### The ablation of *Nat10* results in reduced *Bag3* mRNA stability

**Fig. 6:**
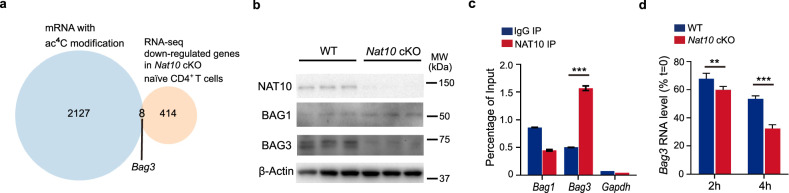
Characterization of NAT10’s role in stabilizing *Bag3* mRNA. **a** A Venn diagram illustrates the overlap of RNA-seq upregulated genes in *Nat10* cKO-derived naïve CD4^+^ T cells compared to WT-derived naïve CD4^+^ T cells depicted in Fig. 5a with the set of ac^4^C-modified genes derived from previously published ac^4^C-RNA immunoprecipitation (RIP) data^11^. **b** Validation of the differential expression of BAG1 and BAG3 proteins by western blot analysis in WT versus *Nat10* cKO naïve CD4^+^ T cells. **c** NAT10 RIP-qPCR assay was applied to detect NAT10-*Bag3* gene interaction in naïve CD4^+^ T cells (*n* = 3). **d** Kinetic analysis of *Bag3* mRNA decay in WT and *Nat10* cKO naïve CD4^+^ T cells, following incubation with actinomycin D for 0, 2, and 4 h (*n* = 3). The residual mRNA levels were normalized to the initial levels at the 0-h time point to assess the stability of *Bag3* mRNA transcripts. Data represent one of three independent experiments and are shown as mean ± SEM. Statistical significance was determined using unpaired Student’s *t*-test: **p < 0.01, ***p < 0.001. NS, not significant.

Extensive research has established that *Nat10*’s role on mRNA molecules is primarily to augment their stability and enhance their translational efficiency^12,14,15^. It follows that the genes that were upregulated in *Nat10* KO naïve CD4^+^ T cells are unlikely to be direct targets of *Nat10*. To elucidate how *Nat10* modulates T cell apoptosis, our investigation pivoted to the downregulated genes, seeking those that might be directly influenced by *Nat10*. We embarked on a comparative analysis, overlapping our RNA-seq data with previous ac^4^C-seq results^12^, which led us to identify a potential anti-apoptotic target: *Bag1* and *Bag3* (Fig. 6a). Both *Bag1* and *Bag3* are recognized for their anti-apoptotic properties, and they possess ac^4^C modification sites on their mRNAs. However, by using qPCR and western blot, we confirmed that while *Bag1* mRNA and protein expression remained largely unchanged in *Nat10*-deficient CD4^+^ T cells, *Bag3* expression was notably diminished (Figs. 5d and 6b).

To substantiate that *Bag3* is a direct target of *Nat10*, we conducted RNA immunoprecipitation (RIP) experiments. These experiments demonstrated that *Bag3* mRNA could be significantly enriched through the immunoprecipitation of the NAT10 protein (Fig. 6c), providing evidence of a direct interaction between NAT10 protein and *Bag3* mRNA. Further, to decipher the mechanism by which *Nat10* regulates *Bag3* expression, we conducted RNA decay experiments. In isolated naïve CD4^+^ T cells, the transcriptional arrest induced by Actinomycin D allowed us to observe that the degradation rate of *Bag3* mRNA in *Nat10* KO CD4^+^ T cells was markedly faster compared to that in WT cells (Fig. 6d). This finding suggests that *Nat10* promotes the stability of *Bag3* mRNA, thereby curbing excessive apoptosis in naïve CD4^+^ T cells and ultimately maintaining T cell homeostasis and pathogenicity (Supplementary Fig. S6).

## Discussion

The immune system functions as a tightly regulated network, relying on precise gene expression control for effective operation. T cells, a vital component of the adaptive immune response, maintain immune homeostasis through a delicate balance of proliferation, differentiation, and apoptosis. Any disruption in their development or functional execution can significantly impact immune surveillance. In this work, our findings reveal that NAT10 deficiency causes a notable disturbance in T cell development under steady-state conditions and mitigates T cell pathogenicity during IBD. This finding is pivotal as it underscores NAT10’s previously unrecognized role in sustaining T cell homeostasis and functionality through RNA ac^4^C modification.

Our previous research, in conjunction with others, has established that RNA modifications such as m^1^A and m^6^A are crucial for maintaining T cell homeostasis and pathogenicity^5–11^. However, these studies have primarily focused on RNA methylation, resulting in a significant gap in knowledge regarding alternative RNA modification mechanisms. NAT10, with its unique role as the sole known enzyme for ac^4^C modification, has garnered substantial interest. This enzyme is implicated in various aspects of RNA metabolism, yet its specific function in preserving T cell functionality has remained elusive and controversial. A recent study observed a significant reduction in the RNA expression of NAT10 in CD4^+^ T cells from systemic lupus erythematosus (SLE) patients compared to healthy controls, along with a decrease in ac^4^C modification^28^. This suggests that NAT10-mediated ac^4^C modification may suppress the onset and progression of SLE. In contrast, our bioinformatics analysis of single-cell data from inflamed and non-inflamed colonic tissues of IBD patients showed that higher NAT10 expression in CD4^+^ T cells correlates with increased inflammatory activity, indicating a potential role for NAT10 in promoting IBD progression. These different observations highlight the complexity of ac^4^C in regulating T cell function.

While previous work has shown a potential correlation between NAT10 expression in CD4^+^ T cells and SLE onset, it lacked direct in vivo evidence^28^. Our study illuminates the essential role of NAT10 in maintaining in vivo T cell homeostasis and its influence on the pathogenicity of CD4^+^ T cells in inducing IBD. Specifically, we observed that the deletion of *Nat10* leads to significant disruptions in T cell homeostasis, characterized by heightened cytokine secretion, increased proliferation, and elevated apoptosis, suggesting a hyperactivated state in T cells, aligning with a recent study that indicates mice with T cell-specific NAT10 deficiency display reduced numbers of mature T cells in peripheral lymphoid organs^29^. However, their study emphasizes NAT10’s role in T cell activation and proliferation through acetylating RACK1 to affect cellular metabolism, while our study focuses on NAT10’s function in epitranscriptomics, with a particular focus on the ac^4^C modification of RNA related to apoptosis regulation. Mechanistically, our RNA-seq analysis revealed that differentially expressed genes were predominantly involved in apoptosis-related pathways, with the anti-apoptotic gene *Bag3* playing a key role in NAT10-mediated T cell survival. The downregulation of *Bag3* in the absence of NAT10 triggers a cascade leading to heightened apoptosis. Bag3, known to inhibit cell death by interacting with anti-apoptotic proteins such as BCL2 and HSP70, is a well-established regulator of apoptosis^30–37^. Our research indicates that NAT10’s effect on *Bag3* mRNA stability is critical for controlling T cell survival.

In conclusion, our study provides new insights into the role of NAT10 as an RNA cytidine acetyltransferase in safeguarding T cell balance through the NAT10-ac^4^C-*Bag3* axis, offering a novel perspective on post-transcriptional regulation in inflammatory diseases. This has profound implications for advancing our understanding of T cell biology and immune regulation. Further studies will be necessary to understand the molecular mechanisms underlying these effects fully and to explore the potential therapeutic implications of modulating NAT10 activity in T cell-related inflammatory diseases. As our exploration into NAT10’s immune system function is still in its infancy, additional studies are crucial for a complete understanding of its extensive impact on immune processes and its potential application in therapeutic strategies.

## Materials and methods

### Mice

*Nat10*^flox/flox^ mice were genetically engineered using the CRISPR/Cas9 system, which involved the insertion of two loxP sites flanking the exon 4. *Cd4*^Cre^ mice were procured from the Jackson Laboratory, *Nat10* ^flox/flox^ mice were crossed with *Cd4*^Cre^ mice to produce *Nat10* ^flox/flox^ *Cd4*^Cre^ offspring lacking NAT10 expression in CD4^+^ T cells, with *Nat10* ^flox/flox^ mice serving as WT controls. All mice were raised and maintained in a specific pathogen-free environment at Cyagen Co., Ltd. Both male and female mice aged 8–12 weeks were utilized in experiments. Animals were randomly assigned to experimental groups, with each cage containing mice from different groups. The Institutional Animal Care and Use Committee (IACUC) of Shanghai Jiao Tong University School of Medicine approved all animal procedures (No. JUMC2023-086-A).

### Cell isolation

The thymus, spleen, and lymphoid node were pressed through a fine mesh of 200-gauge. To obtain splenic single-cell leukocyte suspensions, the erythrocytes were lysed using a red cell lysis buffer from ThermoFisher Scientific.

### Adoptive transfer colitis model

Naïve CD4^+^ T cells were isolated from spleens utilizing the EasySep Mouse Naïve CD4^+^ T Cell Isolation Kit (STEMCELL Technologies). These cells were subsequently labeled with CellTrace Violet (ThermoFisher Scientific). Subsequently, 5 × 10^5^ naïve CD4^+^ T cells from *Nat10* ^flox/flox^ *Cd4*^Cre^ mice and their WT littermate controls were transferred into *Rag1*^–/–^ mice, respectively. Body weight measurements of the recipient mice were taken weekly.

### T cell isolation and stimulation

Naïve CD4^+^ T cells were purified using the EasySep Mouse Naïve CD4^+^ T Cell Isolation Kit (STEMCELL Technologies) following the provided instructions. Subsequently, the purified T cells were stimulated in replicate wells of a 96-well plate using plate-bound anti-CD3 (2 μg/mL) and anti-CD28 (2 μg/mL) antibodies, and cultured in RPMI with 10% fetal bovine serum (TransGen Biotech), 1% streptomycin/penicillin, β-mercaptoethanol (40 nM) and IL-2 (2 ng/mL, ThermoFisher Scientific) for different time points.

### Antibody staining and flow cytometry

Monoclonal antibodies (mAbs) specific to CD3 (clone 145-2C11), CD4 (clones RM4-5, RM4-4, GK1.5), CD8 (clone 53-6.7), CD25 (clone 3C7), CD44 (clone IM7), CD62L (clone MEL-14), CD45 (clone 30-F11), CXCR3 (clone CXCR3-173), IFN-γ (clone XMG1.2), Granzyme B (clone, Granzyme B), Ki67 (clone 16A8), and TCR-β (clone H57-597) were purchased from BioLegend. The Fas antibody (clone Jo2) was purchased from BD Biosciences. The cells were incubated with the antibodies (diluted 1:200) at 4 °C for 30 min to facilitate staining. For intracellular cytokine staining, the cells were first stimulated with a combination of phorbol myristate acetate (50 ng/mL; Sigma), ionomycin (1 μg/mL; Sigma), and GolgiPlug (1 μL/mL; BD Biosciences) for a duration of 4 h. Following surface staining with antibodies, the cells were fixed and permeabilized using a Fixation/Permeabilization Solution Kit from BD Biosciences. Subsequently, the intracellular molecules were stained with specific antibodies. For intracellular transcription factor staining, the cells were fixed and permeabilized using the FOXP3/Transcription Factor Buffer Set provided by BioLegend. The resulting data were collected using a BD Fortessa X20 flow cytometer (BD Biosciences) and analyzed using FlowJo software.

### Apoptosis assay

The PE or APC Annexin V Apoptosis Detection Kit, along with 7-AAD (BioLegend), was utilized to assess the apoptotic status of the cells.

### RNA library preparation, sequencing, and differentially expressed genes analysis

Naïve CD4^+^ T cells from *Nat10*^*f*lox/flox^ *Cd4*^Cre^ and *Nat10*^flox/flox^ mice were isolated using the EasySep Mouse CD4^+^ T Cell Isolation Kit. Total RNA was extracted by the TRIzol reagent (Invitrogen) according to the manufacturer’s protocol. RNA purity and quantification were assessed by NanoDrop 2000 spectrophotometer (ThermoFisher Scientific, USA), and the integrity was evaluated by Agilent 2100 Bioanalyzer (Agilent Technologies, USA). RNA libraries were prepared with NEBNext^®^ Ultra™ II Directional RNA Library Prep Kit for Illumina (NEB) according to the manufacturer’s instructions.

The libraries were sequenced on Illumina HiSeq X Ten platform (150 bp paired-end reads) by Shanghai Neo-Biotechnology Co., Ltd (Shanghai, China). About 50 million raw reads for each sample were generated. After processing raw reads by Trimmomatic, the high-quality clean reads were mapped to the mouse genome (GRCm38.p6) using HISAT2^38^. FPKM^39^ of each gene was calculated by Cufflinks ^40^, and the read counts of each gene were obtained by HTSeq-count^41^. The DESeq2^42^ was used to normalize the raw counts and identify differentially expressed genes (DEGs; |fold change| ≥ 2; *p* < 0.05). Volcano plots and bar plots were performed using the *R* package. KEGG pathway enrichment analysis of DEGs was performed using the phyper function from *R* package “stats” based on the hypergeometric distribution. Two independent biological replicates were performed for RNA Sequencing.

### RT-qPCR

Naïve CD4^+^ T cells were subjected to Total RNA isolation using the TRIzol reagent (Invitrogen), strictly adhering to the manufacturer’s guidelines. Subsequently, the isolated RNA was reverse-transcribed utilizing the HiScript II 1st Strand cDNA Synthesis Kit (Vazyme). For real-time PCR, ChamQ Universal SYBR qPCR Master Mix (Vazyme) was employed. Additionally, *Actb* served as an internal control for the quantification of relative mRNA amounts. The primer sequences utilized for qPCR are enumerated as follows: *Actb* (forward, 5′-GCCAACCGTGAAAAGATGAC-3′; reverse, 5′-CATCACAATGCCTGTGGTAC-3′), *Bax* (forward, 5′-TGAAGACAGGGGCCTTTTTG-3′; reverse, 5′-AATTCGCCGGAGACACTCG-3′), *Dapk2* (forward, 5′-CTCGATGAGGAGCCCAA-ATATG-3′; reverse, 5′-AATTCGCCGGAGACACTCG-3′), *Cdk1* (forward, 5′-AGAAGGTACTT-ACGGTGTGGT-3′; reverse, 5′-GAGAGATTTCCCGAATTGCAGT-3′), *Il12b* (forward, 5′-TGGT-TTGCCATCGTTTTGCTG-3′; reverse, 5′-ACAGGTGAGGTTCACTGTTTCT-3′), *Il17a* (forward, 5′-TTTAACTCCCTTGGCGCAAAA-3′; reverse, 5′-CTTTCCCTCCGCATTGACAC-3′), *Il18* (forward, 5′-GACTCTTGCGTCAACTTCAAGG-3′; reverse, 5′-CAGGCTGTCTTTTGTCAACG-A-3′), *Ifng* (forward, 5′-ATGAACGCTACACACTGCATC-3′; reverse, 5′-CCATCCTTTTGCCAG-TTCCTC-3′), *Tnfsf4* (forward, 5′-AATCTGGAAAACGGATCAAGGC-3′; reverse, 5′-CAGGCA-GACATAGATGAAGCAC-3′), *Bag3* (forward, 5′-CTGGGAGATCAAAATCGACCC-3′; reverse, 5’-GCTGAAGATGCAGTGTCCTTAG-3′), *Bag1* (forward, 5′’-GGAGGAAATGGAAACACCC-3′; reverse, 5′-AGTCTTGGACAACTGGCTCAC-3′), *Gapdh* (forward, 5′-GAGAGTGTTTCCTCGT-CCCG-3′; reverse, 5′-GCCTCACCCCATTTGATGTTAG-3′).

### RNA degradation assay

RNA degradation assay was performed following previously established protocols ^6^. Naïve CD4^+^ T cells were isolated using the EasySep Mouse CD4^+^ T Cell Isolation Kit and seeded into a 96-well plate with a cell density of 5 × 10^5^ cells per well. Actinomycin-D (Sigma-Aldrich) was then added to each well, achieving a final concentration of 5 μM. Subsequently, CD4^+^ T cells were collected at 2 and 4 h post-addition of actinomycin-D. The harvested cells were processed according to the protocol outlined in the “RT-qPCR” section, and the resulting data were normalized to the *t* = 0 time point for comparison.

### Western blot

Naïve CD4^+^ T cells from spleen and lymphoid nodes were purified by EasySep Mouse CD4^+^ T Cell Isolation Kit (Stemcell). The total protein from CD4^+^ T cells was extracted by RIPA lysis buffer (Beyotime, P0013B) supplemented with protease inhibitors (Roche, 11697498001). The primary antibodies used were as follows: NAT10 (Abcam, ab194297, 1:1000); BAX (Cell Signaling Technology, 2772, 1:1000); DAPK2 (Abclonal, A8199, 1:1000); IFN-γ (Abclonal, A12450, 1:1000); BAG1 (Abcam, ab32109, 1:500); BAG3 (Abcam, ab92309, 1:1000). They were diluted in 5% no-fat milk buffer and incubated at 4 °C overnight. After washing the membrane with 0.1% TBST buffer three times, the HRP-conjugated secondary antibody was added to the membrane and incubated at room temperature for 1 h. The final signal was detected by SuperFemto ECL Chemiluminescence Kit (Vazyme) using ChemiDoc MP Imaging System (Biorad), and ACTB (Cell Signaling Technology, 3700, 1:5000) was used as the internal control.

### ATAC-seq

For each sample, 30,000 naïve CD4^+^ T cells from WT and *Nat10* cKO mice with 90% viability were spun down for cell pellet collection. After being resuspended in nuclear lysis buffer, the pellets were collected and treated with the TruePrepTM DNA Library Prep Kit V2 (Vazyme, TD501) following the manufacturer’s instructions. PCR was used to amplify the transposed DNA fragments using primers with different Barcodes. Illumina Hiseq X ten (PE150) was used to sequence the library. Bowtie 2 (version 2.2.3) was used to map the data to the Mus Musculus (mm10) reference genome, and MACS2 (version 2.1.1) was used to identify binding sites with the parameter ‘-q 0.05–nomodel–extsize 150–keep-dup all–call-summits’.

### RIP-qPCR

Naïve CD4^+^ T cells were lysed in polysome lysis buffer (PLB, 0.5% NP40, 100 mM KCl, 5 mM MgCl_2_, 10 mM HEPES, 1 mM DTT, 100 units/mL RNase inhibitor, and 1× protease inhibitor cocktail, pH7.0) by mechanical homogenizer and centrifuged at 20,000× *g* for 20 min at 4 °C to remove the debris. The supernatant was pre-cleared with blocked Dynabeads and immunoprecipitated with antibody-conjugated (Proteintech, 13365-1-AP) or normal IgG-conjugated Dynabeads at 4 °C for at least 3 h. Next, the beads were washed four times with NT2 buffer (0.05% NP40, 50 mM Tris-HCl, 150 mM NaCl, and 1 mM MgCl_2_, pH 7.4) supplemented with RNase and proteinase inhibitor. Before the last washing, the beads were divided into two portions. A small portion was used to isolate protein for enrichment identification of target protein, the remaining portion was resuspended in 100 μL of NT2 buffer supplemented with RNase inhibitor and 30 μg of proteinase K to release the RNA at 55 °C for 30 min, and the RNA was eluted using 1 mL of TRIzol.

### Statistical analysis

To compare pairs of groups, we employed unpaired Student’s *t*-test and two-way analysis of variance (ANOVA). All the collected data are represented as the mean ± standard error of the mean (SEM). Statistically significant differences were determined when the values of *p* < 0.05.

## Supplementary information

Supplementary Figs. S1–S6

## Data Availability

The RNA sequencing data obtained from naïve CD4^+^ T cells of WT and *Nat10* conditional knockout mice have been deposited in the Gene Expression Omnibus (GEO) database with the accession number GSE284711. The ATAC-seq data obtained from naïve CD4^+^ T cells of WT and *Nat10* cKO mice have been deposited in the Gene Expression Omnibus (GEO) database with the code GSE285309. Other data that support the findings of this study are available from the corresponding author upon reasonable request. Source data are provided in the manuscript.
