# Supplementary material for: RNA cytidine acetyltransferase NAT10 maintains T cell pathogenicity in inflammatory bowel disease
Source: Cell Discov. 2025 Mar 4;11:19. doi: 10.1038/s41421-025-00781-5 (PMC11880361; doi:10.1038/s41421-025-00781-5)
Supplement: Supplementary file 1 — Supplementary Figs. S1–S6 [file 41421_2025_781_MOESM1_ESM.pdf]

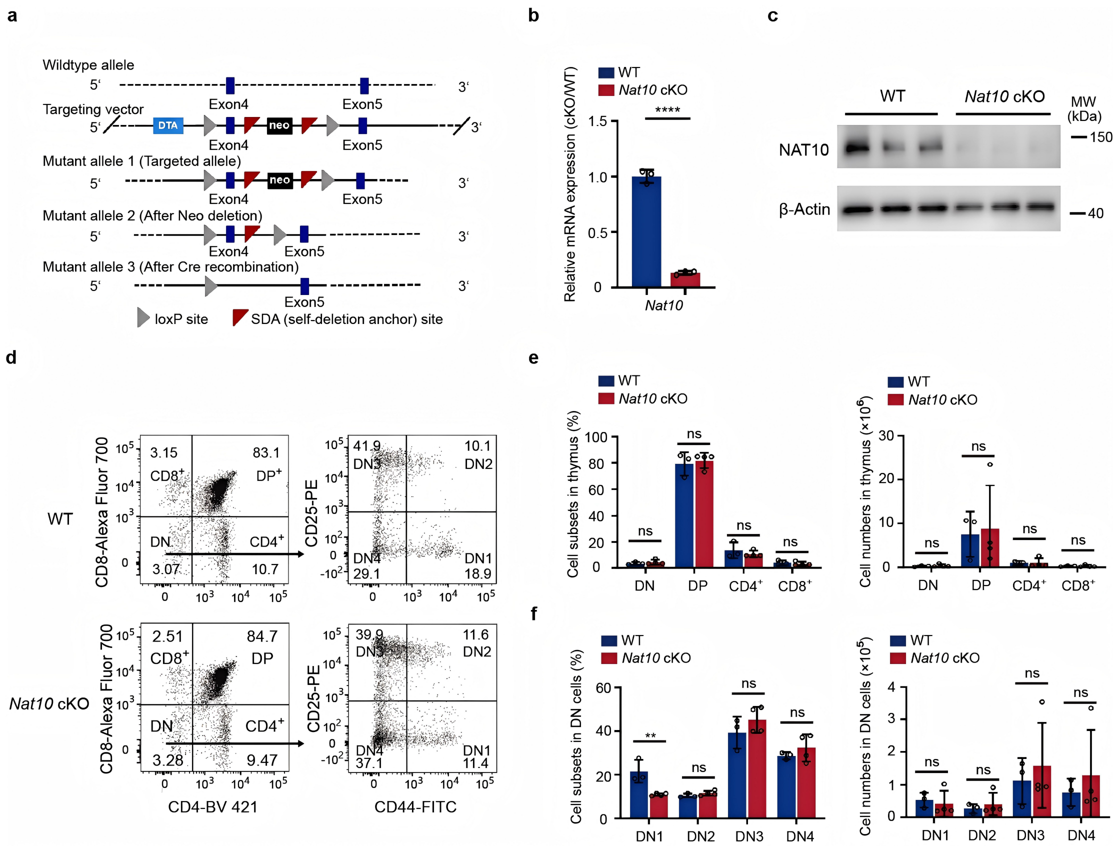

Supplementary Fig. S1 *Nat10* is not essential for T cell development. a Strategy for targeting the fourth intron of *Nat10* gene. **b** qRT-PCR analysis of *Nat10* transcripts in exon 4 of WT and *Nat10*-deficient naïve CD4$^+$ T cells, normalized to *Actb* (n=3). **c** Protein expression level of NAT10 was analyzed by Western blot in splenic naïve CD4$^+$ T cell isolated from wild-type (WT) and *Nat10* conditional knockout (cKO) mice. **d** Dot plots illustrate the composition of CD4$^+$ and CD8$^+$ T cell subsets and different Double negative (DN) stages in the thymus from WT and *Nat10* cKO mice. **e-f** Absolute counts and relative proportions of T cell subsets (**e**) and different DN stages (**f**) in the thymus are presented (n=3-4). Data represent one of three independent experiments and are shown as mean ± SEM. Statistical significance was determined using unpaired Student's *t*-test: $^{**}p < 0.01$, $^{****}p < 0.0001$. NS, not significant.

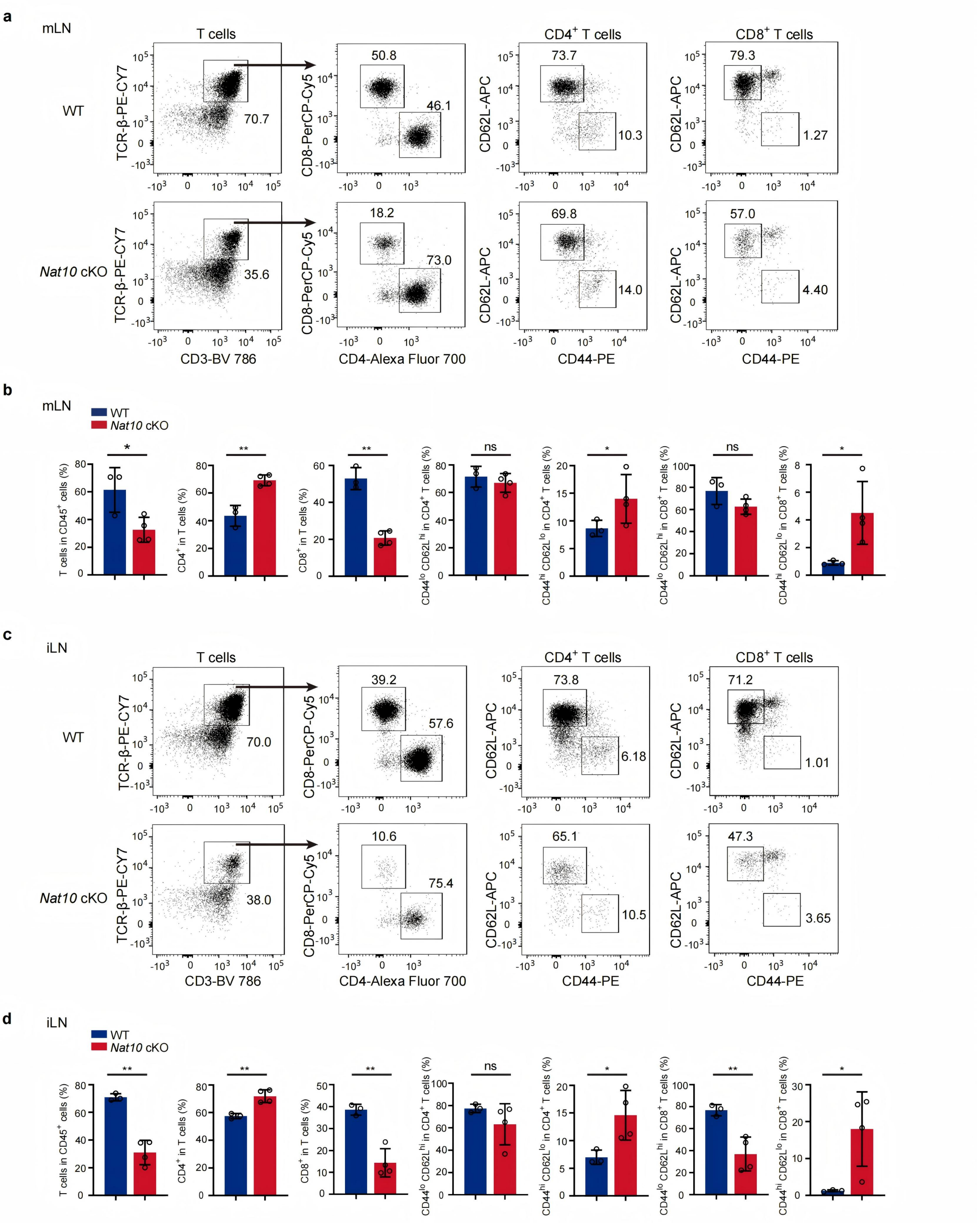

Supplementary Fig. S2 T cell-specific deletion of *Nat10* leads to disrupted T cell homeostasis in peripheral lymph nodes. a Representative dot plots illustrate the percentages of CD4^{+} and CD8^{+} T cells in the mesenteric lymph nodes (mLNs) of WT and *Nat10* cKO mice. Expression of CD62L and CD44 on T cell subsets is also shown. Numbers in the quadrants indicate the percentage of cells in each subset. **b** The composition of various T cell subsets and the expression of CD62L and CD44 on T cell subsets in the mLN of WT and *Nat10* cKO mice are quantified (n=3-4). **c** Representative dot plots illustrate the percentages of CD4^{+} and CD8^{+} T cells in the inguinal lymph nodes (iLNs) of WT and *Nat10* cKO mice. Expression of CD62L and CD44 on T cell subsets is also shown. Numbers in the quadrants

indicate the percentage of cells in each subset. **d** The composition of various T cell subsets and the expression of CD62L and CD44 on T cell subsets in the iLN of WT and *Nat10* cKO mice are quantified (n=3-4).Data represent one of three independent experiments and are shown as mean ± SEM. Statistical significance was determined using unpaired Student's t-test: $^*p < 0.05$, $^{**}p < 0.01$. NS, not significant.

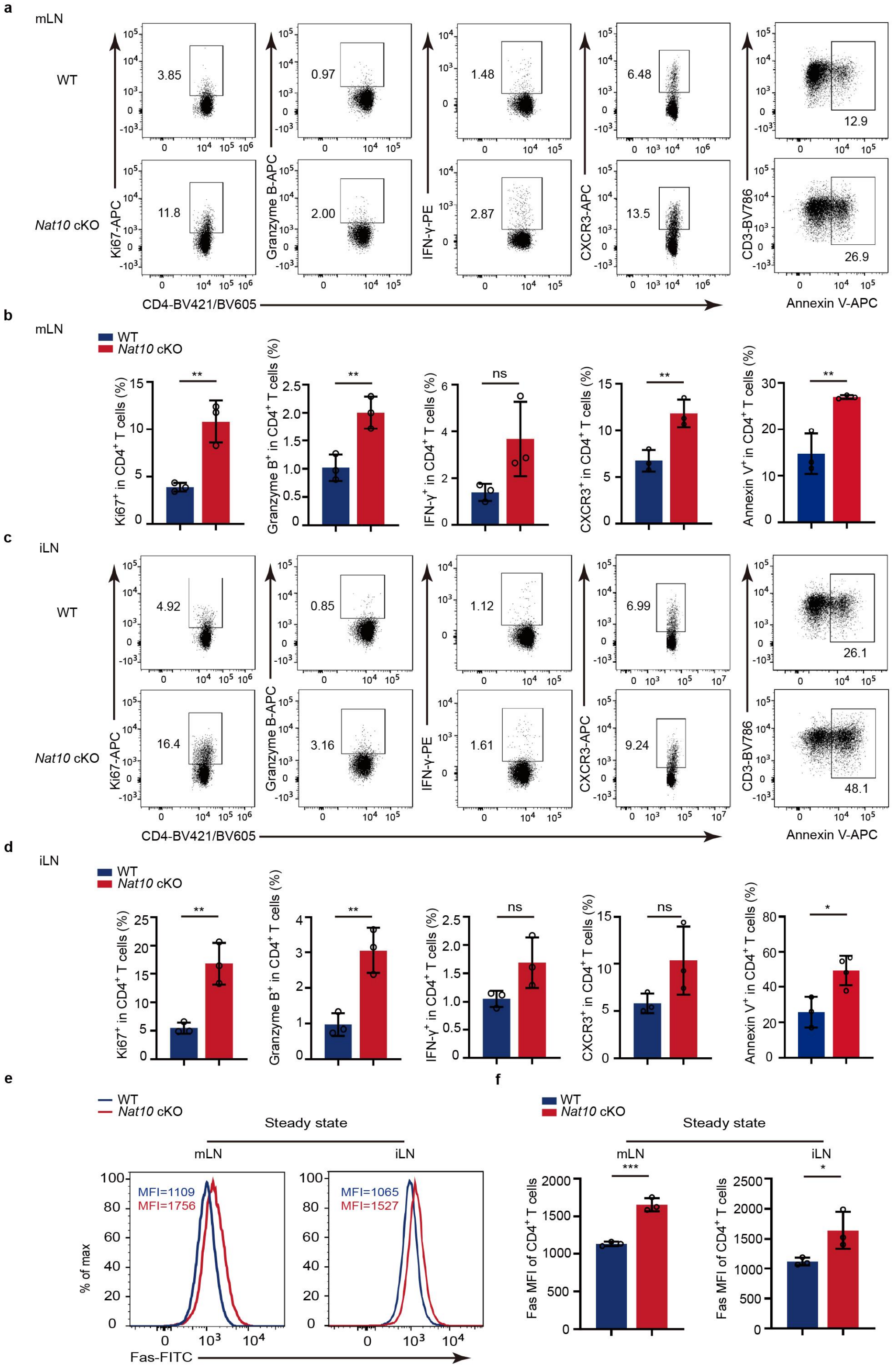

Supplementary Fig. S3 ***Nat10*-deficient naïve CD4^{+} T cells show hyperactivation and increased apoptosis in peripheral lymph nodes. a** Characteristic dot plots illustrate the

distribution of Ki67, Granzyme B, IFN-γ, CXCR3 and Annexin V-positive CD4$^+$ T cells in the mLN of WT and *Nat10* cKO mice at a steady state. **b** Percentages of Ki67, Granzyme B, IFN-γ, CXCR3 and Annexin V-positive CD4$^+$ T cells in **a** are shown (n = 3). **c** Characteristic dot plots illustrate the distribution of Ki67, Granzyme B, IFN-γ, CXCR3 and Annexin V-positive CD4$^+$ T cells in the iLN of WT and *Nat10* cKO mice at a steady state. **d** Percentages of Ki67, Granzyme B, IFN-γ, CXCR3 and Annexin V-positive CD4$^+$ T cells in **c** are shown (n = 3). **e** Characteristic flow cytometry histograms illustrate the distribution of Fas-positive CD4$^+$ T cells in the mLN and iLN from WT and *Nat10* cKO mice at a steady state. **f** Percentages of Fas mean fluorescence intensity (MFI) of CD4$^+$ T cells in **e** are shown (n = 3). Data represent one of three independent experiments and are shown as mean ± SEM. Statistical significance was determined using unpaired Student's *t*-test: *$p < 0.05$, **$p < 0.01$, ***$p < 0.001$. NS, not significant.

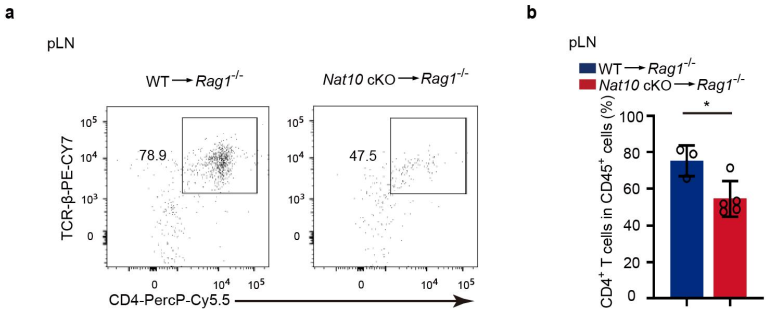

Supplementary Fig. S4 Characterization of transferred CD4$^+$ T cells from WT and *Nat10* cKO mice in the peripheral lymph node of *Rag1$^{-/-}$* recipients. **a** Naïve CD4$^+$ T cells (5×10^5) were purified from either *Nat10* cKO mice or their WT counterparts and then transferred into *Rag1*$^{-/-}$ mice. Characteristic dot plots illustrate the composition of transferred CD4$^+$ T cells in the peripheral lymph node (pLN) of recipient mice 10 weeks post-transfer. **b** The proportion of CD4$^+$ T cells in **a** (n =3-5) are shown. Data represent one of three independent experiments and are shown as mean ± SEM. Statistical significance was determined using unpaired Student's *t*-test: *$p < 0.05$. NS, not significant.

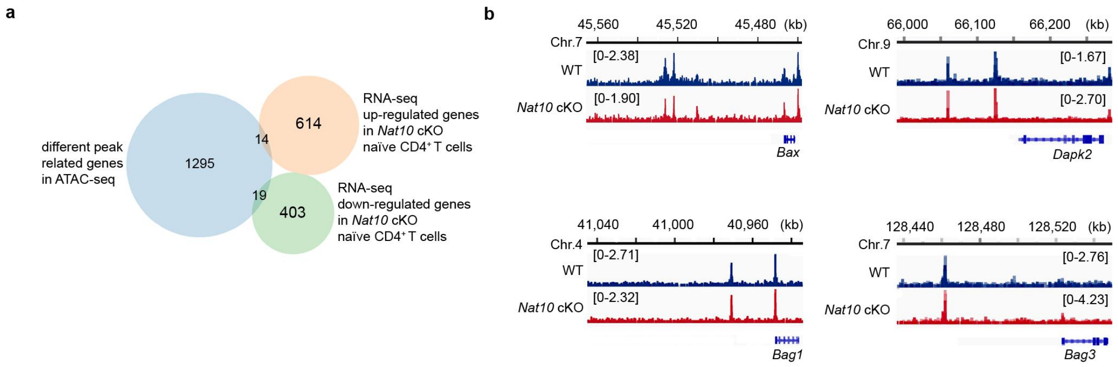

Supplementary Fig. S5 Comparative analysis of chromatin accessibility between WT and *Nat10* KO naïve CD4$^+$ T cells. a A comparison of genes that overlap between the RNA-seq and ATAC-seq data. The RNA-seq data are from Fig. 5a, and the different peak-related genes identified in the ATAC-seq data are derived from *Nat10* cKO -derived naïve CD4$^+$ T cells compared to WT-derived naïve CD4$^+$ T cells. **b** The ATAC-seq results indicate no significant differences in chromatin accessibility between WT and *Nat10*-deficient naïve CD4$^+$ T cells for the genes *Bax*, *Dapk2*, *Bag1*, and *Bag3*.

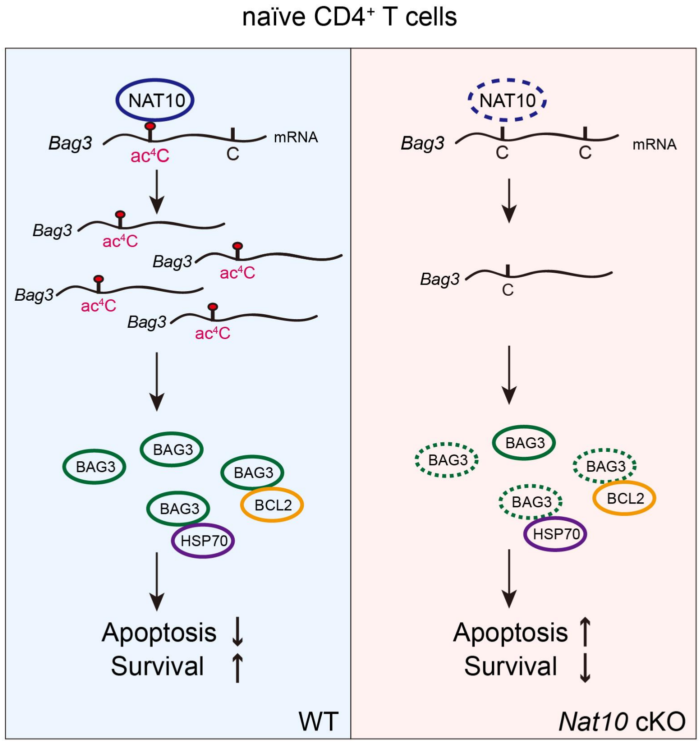

Supplementary Fig. S6 Schematic diagram of the role of NAT10-ac^{4}C-*Bag3* in regulating T cell apoptosis.
